# Perceived competence and cognitive bias in nurses' assessment of intimate partner violence: a cross-sectional study

**DOI:** 10.3389/fpubh.2026.1835799

**Published:** 2026-05-07

**Authors:** David Casero-Benavente, Natalia Mudarra-García, Guillermo Charneco-Salguero, Francisco Lencina-Navarro, Diego Pérez-Iglesias, Cecilia Castillo-Gallardo, Raquel Vázquez-Royo, Paula Panadero-Puente, Francisco Javier García-Sánchez, José Miguel Cárdenas-Rebollo

**Affiliations:** 1 Nursing Department, University San Pablo-CEU, CEU Universities, Boadilla del Monte, Spain; 2 Emergency Room Service, Instituto de Investigación Puerta de Hierro Segovia Arana, Department of Medicine (University Complutense of Madrid), Hospital Universitario Infanta Cristina, Parla, Madrid; 3 Department of Mathematics and Data Science, Faculty of Economics and Business Sciences, University San Pablo-CEU, CEU Universities, Madrid, Spain

**Keywords:** cognitive bias, domestic violence detection, healthcare professionals, intimate partner violence, nursing, training

## Abstract

**Introduction:**

Intimate partner violence (IPV) represents a complex social and health problem that requires multidisciplinary responses. Healthcare professionals, particularly nurses, play a key role in the identification and management of IPV cases. However, limited training and potential cognitive biases may affect professional assessment and intervention.

**Methods:**

A cross-sectional descriptive study was conducted among 202 nursing professionals in Spain. Perceived competence in IPV management was evaluated using the validated Intimate Partner Violence Competency Scale for Nurses (ECVPE), which assesses four dimensions: intervention and referral, detection and assessment of abuse, documentation and record-keeping, and psychosocial support. Descriptive statistics, Student's *t*-tests, and one-way ANOVA were used to analyze differences according to sociodemographic variables.

**Results:**

The lowest levels of perceived competence were observed in the intervention and referral dimension (M = 2.45), while the highest scores were found in psychosocial support (M = 3.36). Professional experience was the only variable significantly associated with perceived competence in the dimensions of detection and documentation of abuse. Additionally, most participants conceptualized IPV primarily as violence against women in heterosexual relationships, indicating the presence of perceptual biases that may influence professional assessment.

**Conclusions:**

Nursing professionals reported heterogeneous levels of perceived competence in IPV management, with notable gaps in intervention and referral processes. The results highlight the need for training strategies that strengthen professional competencies while addressing implicit biases in the interpretation of violence within intimate relationships.

## 1 Introduction

Intimate partner violence (IPV) is recognized as a complex social and health problem with significant psychological, physical, and social consequences for individuals and families. IPV occurs within the context of intimate relationships and may affect individuals regardless of gender, sexual orientation, or relationship structure. In recent decades, research on violence within intimate relationships has expanded considerably; however, much of the literature has traditionally focused on gender-based violence against women. While this perspective has been crucial for advancing awareness and protection policies, it may also contribute to conceptual ambiguities that affect the recognition of other forms of violence within intimate relationships (1, 2).

**Figure 1:**
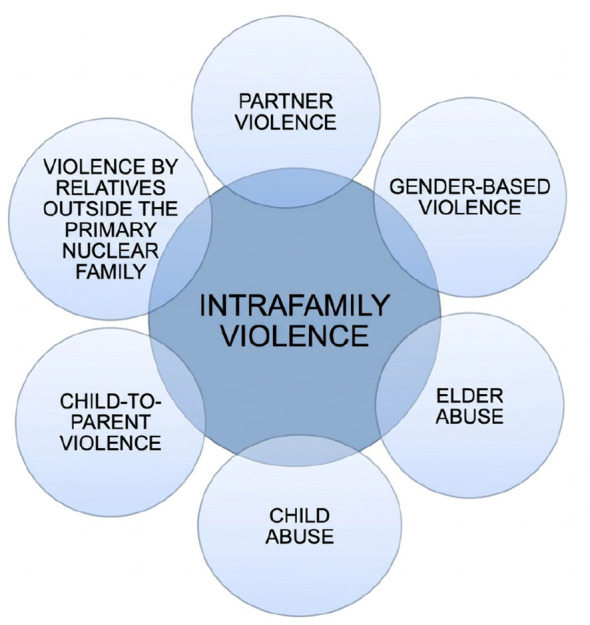
Main types of intrafamily violence considered within the conceptual framework of the study.

Healthcare professionals frequently encounter victims of intimate partner violence, often before the situation becomes visible to legal or social services. For this reason, healthcare settings represent an important opportunity for early detection and intervention. Among healthcare professionals, nurses are in a particularly significant position due to their continuous contact with patients and their role in holistic care. Their responsibilities may include identifying potential signs of abuse, documenting clinical findings, providing emotional support, and referring victims to appropriate services. Nevertheless, previous research indicates that many healthcare professionals feel insufficiently prepared to address IPV situations in clinical practice, often reporting uncertainty, lack of training, and difficulties in managing sensitive conversations with patients (3–5). The emotional impact of caring for victims of violence and the lack of institutional support may also influence professionals' willingness to intervene, further complicating the clinical management of these situations (6). The conceptual framework adopted in this study considers intimate partner violence as part of the broader spectrum of intrafamily violence. The main categories of intrafamily violence considered in this framework are summarized in Figure 1.

**Figure 2:**
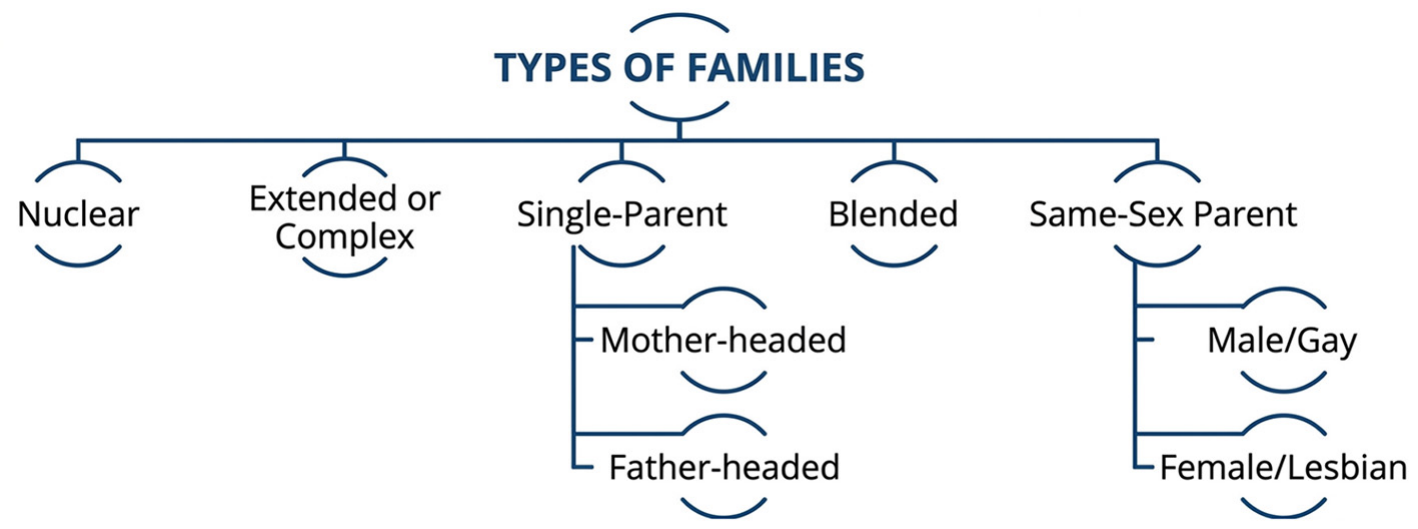
Overview of family structures and relationship configurations considered relevant to the conceptual understanding of intimate partner violence.

Another important challenge in the professional assessment of intimate partner violence relates to the presence of stereotypes and cognitive biases regarding the typical profiles of victims and perpetrators. The traditional representation of IPV frequently assumes a heterosexual relationship in which the man is the aggressor and the woman is the victim (7). Although this pattern is highly prevalent and well documented, it does not fully reflect the diversity of violence dynamics that may occur within intimate relationships (8–10). Studies exploring violence perpetrated by women toward male partners suggest that motivational patterns and psychological characteristics may be comparable to those found in male perpetrators, although certain forms of violence, such as sexual violence, remain less frequent (11, 12). Furthermore, the increasing recognition of diverse family structures and sexual orientations highlights the importance of adopting inclusive perspectives when assessing IPV situations (13, 14). The increasing diversity of family and relationship structures reinforces the need for inclusive approaches to the assessment of intimate partner violence. A conceptual overview of family structures relevant to this perspective is provided in Figure 2.

The presence of stereotypes or conceptual confusion in the interpretation of IPV may have important implications for professional practice. If healthcare professionals approach IPV situations based on preconceived models of violence, certain cases may remain undetected or be incorrectly interpreted. In addition, inadequate documentation of suspected abuse may have relevant legal implications, particularly when medical records are later used in judicial or forensic contexts (15, 16).

Previous frameworks have attempted to systematize nursing care in cases of violence. For example, the SATELLITE model provides a structured approach to assessment and intervention in cases of sexual violence, emphasizing the importance of systematic evaluation and professional preparedness. These approaches highlight the need for standardized tools and training to improve nurses' competence in addressing violence-related cases (17, 18).

Despite the relevance of healthcare professionals in the identification and management of IPV, research evaluating their perceived competencies and preparedness remains limited, particularly in the Spanish context. Understanding how nurses perceive their own training and abilities in key areas such as detection, intervention, documentation, and psychosocial support may help identify gaps in professional preparation and inform the development of targeted training strategies. Therefore, the aim of this study was to assess perceived competence in the management of intimate partner violence and to explore perceptual biases in its conceptualization among nursing rofessionals.

## 2 Methods

### 2.1 Study design

A cross-sectional, descriptive pilot study with a quantitative approach was conducted to evaluate nursing professionals' perceived competence in the management of intimate partner violence (IPV). The study used a structured questionnaire based on a validated scale designed to assess professional competencies related to IPV management within the nursing process.

### 2.2 Participants

The target population consisted of registered nurses working in clinical care settings in the Community of Madrid (Spain). A total of 202 nursing professionals participated in the study. The sample size was calculated to achieve a 95% confidence level with a margin of error below 5%.

Participants were recruited from different healthcare settings, including hospital and primary care services, using a non-probabilistic convenience sampling approach through online dissemination channels.

#### 2.2.1 Inclusion criteria

- Active nurses working in clinical care settings in the Community of Madrid.
- Official nursing qualifications and professional registration in Spain.
- Voluntary participation in the study.

#### 2.2.2 Exclusion criteria

- Nurses not currently engaged in clinical practice.
- Professionals without official professional registration.
- Nursing students who had not yet completed their degree.

### 2.3 Measures

Perceived competence in the management of intimate partner violence (IPV) was assessed using the ECVPE scale (Escala de Competencia en Violencia de Pareja en Enfermería). This instrument evaluates perceived competence through items reflecting routine clinical activities related to IPV management.

The ECVPE scale was conceptually informed by the Nursing Intervention Classification (NIC), particularly intervention 6,403: Intimate Partner Violence, which served as a framework for item development. However, the ECVPE constitutes a distinct measurement instrument.

The scale includes 26 items grouped into four dimensions: (1) Intervention and Referral, (2) Detection and Assessment of Abuse, (3) Documentation and Record-Keeping, and (4) Psychosocial Support.

Responses were recorded on a five-point Likert scale (1 = never to 5 = always), where higher scores indicate greater perceived competence.

The internal consistency of the scale demonstrated excellent reliability, with a Cronbach's alpha of 0.97, while the adequacy of the data for factor analysis was confirmed by a Kaiser–Meyer–Olkin (KMO) value of 0.947 and Bartlett's test of sphericity (*p* < 0.001), supporting the proposed factorial structure of the instrument.

Additionally, three complementary items were included to explore potential perceptual biases regarding the conceptualization of intimate partner violence among healthcare professionals.

### 2.4 Procedure

Data collection was conducted between June 2024 and May 2025 using an anonymous online questionnaire distributed through Microsoft Forms. The survey was disseminated through professional networks, healthcare institutions, professional associations, and social media platforms, providing participants with a direct link and QR code to access the questionnaire.

Participation was voluntary and anonymous. Before accessing the questionnaire, participants received information about the study objectives and were required to provide informed consent electronically.

### 2.5 Instruments

The ECVPE scale includes items derived from standardized nursing activities described in the Nursing Intervention Classification (NIC), ensuring content validity and alignment with clinical practice. Participants were provided with a definition of intimate partner violence prior to completing the questionnaire to ensure conceptual consistency and reduce potential bias.

These items were specifically adapted to assess perceived competence rather than merely the frequency of task performance.

Additionally, three complementary items were included to explore perceptual biases related to the conceptualization of intimate partner violence, including representations of victims, aggressors, and relationship contexts.

The questionnaire was administered in Spanish and the full questionnaire used in this study is provided in Supplementary Appendix A.

### 2.6 Statistical analysis

Data were coded and analyzed using IBM SPSS Statistics version 29.0. Descriptive statistics were calculated to summarize the sociodemographic characteristics of the sample, including sex, age, years of professional experience, workplace setting, and academic level.

For the four dimensions of perceived competence, means, standard deviations, and medians were calculated. To evaluate differences according to sex, Student's t-test for independent samples was applied. Differences across other sociodemographic variables were analyzed using one-way analysis of variance (ANOVA).

Prior to conducting ANOVA, the assumption of homogeneity of variances was tested using Levene's test. When statistically significant differences were identified, post-hoc comparisons (Tukey HSD or Games–Howell) were planned to determine which groups differed significantly. Statistical significance was established at *p* < 0.05.

Assumptions for parametric testing were assessed, including normality and homogeneity of variance. Effect sizes were calculated where appropriate.

### 2.7 Ethical considerations

The study was approved by the Bioethics Committee of CEU San Pablo University (approval code 843/24/104). Participation was voluntary, anonymous, and confidential. All participants provided informed consent prior to completing the questionnaire. The study followed the ethical principles established in the Declaration of Helsinki for research involving human participants.

## 3 Results

### 3.1 Sample characteristics

The study included a total of 202 nursing professionals. The sample was predominantly female, with 165 women (81.7%) and 34 men (16.8%). Regarding professional experience, 24.3% (*n* = 49) had less than five years of experience, 14.9% (*n* = 30) had between six and ten years, 23.8% (*n* = 48) had between eleven and fifteen years, and 37.1% (*n* = 75) had more than sixteen years of professional experience.

In terms of workplace setting, the majority of participants worked in hospital settings (49.0%, *n* = 99) or primary care services (37.1%, *n* = 75). Regarding academic level, 36.6% (*n* = 74) held a nursing diploma, 38.6% (*n* = 78) had a bachelor's degree, 21.8% (*n* = 44) had postgraduate training, and 3% (*n* = 6) held a doctoral degree.

Age distribution showed that 34.2% (*n* = 69) of participants were between 31 and 40 years, followed by 26.7% (*n* = 54) aged 20–30 years, 25.2% (*n* = 51) aged 41–50 years, and 13.9% (*n* = 28) aged over 51 years. The internal consistency of the scale in the present sample was high (Cronbach's α = 0.92).

### 3.2 Descriptive statistics of perceived competence

Perceived competence in intimate partner violence management was evaluated across four dimensions. Descriptive statistics are presented in Table 1.

**Table 1:** Descriptive statistics of the study dimensions.

Dimension	N	Mean	Standard deviation	Median
D1 – Intervention and referral	202	2.45	0.96	2.33
D2 – Detection and assessment of abuse	202	3.07	0.83	3.00
D3 – Documentation and record-keeping	202	3.10	0.83	3.00
D4 – Psychosocial support	202	3.36	0.84	3.33

The results indicate that the dimension with the lowest perceived competence was Intervention and Referral (D1), with a mean score of 2.45 (SD = 0.96). In contrast, the highest perceived competence was observed in the Psychosocial Support (D4) dimension, with a mean score of 3.36 (SD = 0.84).

In terms of specific interventions, activities related to psychosocial support were the most frequently reported, while actions involving formal referral and coordination with external resources were less commonly performed. Detection and assessment activities showed moderate frequency, indicating variability in clinical practice.

The dimensions Detection and Assessment of Abuse (D2) and Documentation and Record-Keeping (D3) showed intermediate levels of perceived competence, with mean scores of 3.07 (SD = 0.83) and 3.10 (SD = 0.83), respectively.

These results suggest that nursing professionals perceive themselves as more competent in providing emotional and psychosocial support than in performing direct interventions or referrals in cases of intimate partner violence.

The questionnaire included perceived competence grouped into four dimensions: intervention and referral, detection and assessment of abuse, documentation and record-keeping, and psychosocial support (see Table 2 and Supplementary Appendix A).

**Table 2:** Items included in the nursing activities assessment scale.

Item	Dimension	Description of nursing activity
1	Detection and assessment of abuse	Assess the patient's safety and risk of further violence.
2	Detection and assessment of abuse	Identify signs and symptoms compatible with intimate partner violence.
3	Detection and assessment of abuse	Explore the patient's perception of their situation and possible abuse.
4	Psychosocial support	Provide emotional support to the patient experiencing violence.
5	Psychosocial support	Encourage the patient to express concerns and fears.
6	Intervention and referral	Inform the patient about available resources and support services.
7	Intervention and referral	Facilitate referral to specialized services when necessary.
8	Documentation and record-keeping	Document findings related to suspected or confirmed abuse.
9	Documentation and record-keeping	Maintain confidentiality and privacy when addressing violence-related issues.
10	Intervention and referral	Collaborate with other healthcare professionals in case management.
11	Psychosocial support	Provide guidance on safety planning when appropriate.
12	Intervention and referral	Offer information about legal and social support resources.
13	Psychosocial support	Support the patient in decision-making related to safety and wellbeing.

### 3.3 Differences in perceived competence based on activity-related items by sex

Differences in perceived competence based on activity-related items between male and female participants were analyzed using Student's *t*-test for independent samples. The results are summarized in Table 3.

**Table 3:** Comparison of study dimensions by sex.

Dimension	Sex	N	Mean	SD	t	*p*-value
D1 – Intervention and referral	Women	165	2.4768	0.96288	0.771	0.442
	Men	34	2.3431	0.96220		
D2 – Detection and assessment of abuse	Women	165	3.0667	0.82327	0.046	0.963
	Men	34	3.0735	0.91673		
D3 – Documentation and record-keeping	Women	165	3.1341	0.84165	0.887	0.376
	Men	34	2.9816	0.77858		
D4 – Psychosocial support	Women	165	3.4162	0.81621	1.944	0.053
	Men	34	3.0931	0.93851		

No statistically significant differences were observed between men and women in any of the four competence dimensions (*p* > 0.05). Mean scores were comparable across both groups in the dimensions of intervention and referral, detection and assessment, documentation and record-keeping, and psychosocial support.

These findings suggest that perceived competence in IPV management among nurses does not appear to differ according to sex.

### 3.4 Differences according to professional experience

A one-way analysis of variance (ANOVA) was conducted to examine potential differences in perceived competence according to years of professional experience.

Statistically significant differences were observed in the dimensions Detection and Assessment of Abuse (D2) (*p* = 0.020) and Documentation and Record-Keeping (D3) (*p* = 0.032). No significant differences were found for the dimensions Intervention and Referral (D1) or Psychosocial Support (D4).

Mean scores for the Detection and Assessment of Abuse dimension were 2.84 for professionals with less than 5 years of experience, 3.35 for those with 6 to 10 years, 2.94 for those with 11 to 15 years, and 3.18 for those with more than 16 years of experience.

Similarly, in the Documentation and Record-Keeping dimension, mean scores were 2.87 for professionals with less than 5 years of experience, 3.42 for those with 6 to 10 years, 2.96 for those with 11 to 15 years, and 3.20 for those with more than 16 years of experience.

These results indicate that nurses with six to ten years of professional experience reported the highest levels of perceived competence in both detection and documentation of IPV cases (Table 4).

**Table 4:** Associations between study dimensions and sociodemographic and professional variables.

Dimension	Years of experience	Age	Workplace setting	Academic level
D1 – Intervention and referral	Not significant	Not significant	Not significant	Not significant
D2 – Detection and assessment of Abuse	Significant (*p* = 0.020)	Not significant	Not significant	Not significant
D3 – Documentation and record-keeping	Significant (*p* = 0.032)	Not significant	Not significant	Not significant
D4 – Psychosocial support	Not significant	Not significant	Not significant	Not significant

### 3.5 Perceptual bias in the conceptualization of intimate partner violence

Additional items included in the questionnaire explored potential perceptual biases regarding the conceptualization of intimate partner violence among participants.

Most respondents reported that, when completing the scale, they had primarily imagined a female victim (83%) and a heterosexual relationship with a male aggressor (82%). Furthermore, when asked about the main barriers to performing nursing interventions in IPV situations, 47% of participants identified difficulties in patient interaction and interviewing skills as the primary obstacle, followed by lack of time (34%).

These findings suggest that stereotypical representations of IPV may influence how healthcare professionals conceptualize violence within intimate relationships.

## 4 Discussion

This study examined perceived competence in IPV management using the ECVPE scale, a multidimensional instrument grounded in clinically relevant nursing activities.

Although the ECVPE scale includes items reflecting clinical activities, it is designed to assess perceived competence as a multidimensional construct. Therefore, the use of activity-based items should not be interpreted as a direct measure of frequency alone, but as an operationalization of competence through practice-oriented behaviors.

These results are consistent with previous studies indicating that healthcare professionals often feel more comfortable providing emotional support to victims than performing direct interventions or initiating referral processes in cases of violence (4, 5). The complexity of IPV situations, combined with the potential legal and ethical implications of intervention, may contribute to professional uncertainty and hesitation when addressing these cases in clinical settings. Additionally, the lack of specific training and standardized protocols has been identified as a significant barrier to effective intervention among healthcare professionals (3).

In contrast, the dimension related to psychosocial support showed the highest levels of perceived competence. This finding may be explained by the traditional emphasis of nursing education on holistic care and emotional support, which may provide professionals with greater confidence in these aspects of patient care. Similar patterns have been observed in previous research examining healthcare professionals' attitudes and competencies regarding violence within intimate relationships (1).

Another relevant finding of the present study was the influence of professional experience on perceived competence in the dimensions of detection and documentation of abuse. Nurses with six to ten years of professional experience reported the highest levels of perceived competence in these areas. This pattern may reflect the progressive acquisition of clinical experience and the consolidation of professional judgment during the early stages of professional development. Previous research has suggested that professional exposure to complex clinical situations contributes to the development of more refined observational and documentation skills among healthcare professionals (19).

These findings are consistent with previous studies highlighting similar patterns in nursing competence and preparedness in IPV management (5, 20).

Interestingly, other sociodemographic variables, including sex, age, workplace setting, and academic level, did not show significant differences in perceived competence. This suggests that the gaps identified in the management of IPV may be structural rather than individual, affecting healthcare professionals across different demographic and professional contexts. Similar findings have been reported in studies analyzing the involvement of healthcare professionals in the detection and management of gender-based violence (21).

Beyond the differences in perceived competence, the results of the additional survey items revealed the presence of perceptual biases in how participants conceptualized IPV. A large proportion of respondents reported that when answering the questionnaire, they had primarily imagined a female victim and a male aggressor in a heterosexual relationship. While this representation corresponds to one of the most prevalent forms of intimate partner violence, it does not fully capture the diversity of violence dynamics that may occur within intimate relationships.

These findings are consistent with previous studies highlighting the importance of structured frameworks and training in improving nurses' competence and confidence in managing violence-related cases. Recent evidence suggests that targeted educational interventions can significantly enhance nurses' preparedness and decision-making in these contexts (22).

Previous research has highlighted that violence within intimate relationships may also occur in same-sex couples or involve male victims, although these situations are often less visible in both research and professional practice (12, 23). The persistence of stereotypical representations of IPV may limit professionals' ability to recognize less visible forms of violence and may contribute to under-detection in certain populations. An inclusive approach that considers diverse family structures and sexual orientations is essential to ensure accurate identification and management of IPV cases (13, 14).

The findings related to perceived barriers to intervention further reinforce these interpretations. Nearly half of the participants identified difficulties in patient interaction and interviewing skills as the main obstacle when addressing IPV cases. These results are consistent with previous studies suggesting that communication challenges and emotional discomfort may hinder professionals' ability to initiate conversations about violence with patients (6). Furthermore, lack of time was identified as another important barrier, reflecting structural limitations within healthcare systems that may restrict opportunities for in-depth patient assessment.

From a professional perspective, the findings of this study highlight the need for structured training programs focused on IPV detection, documentation, and intervention strategies. Training initiatives should not only address clinical competencies but also incorporate components aimed at recognizing and overcoming implicit biases that may influence professional judgment. Previous literature has emphasized that effective training programs combine theoretical knowledge with practical skills and reflective approaches that allow professionals to critically examine their own assumptions and attitudes toward violence (15, 16).

Accurate documentation of suspected IPV cases represents another critical aspect of professional practice. Clinical records may later play an important role in legal or forensic proceedings, making it essential for healthcare professionals to document cases of violence carefully and systematically. Inadequate documentation may not only compromise patient care but also affect the legal protection of victims (15).

Despite the relevance of the findings, several limitations should be considered. First, the sample size of 202 participants, while sufficient for exploratory analysis, may limit the generalizability of the results to other healthcare contexts or regions. Second, the study relied on self-reported perceptions of competence, which may not necessarily reflect actual professional performance in real clinical situations. Finally, the cross-sectional design of the study does not allow causal relationships to be established between professional characteristics and perceived competence.

Future research should consider larger and more diverse samples, including professionals from different healthcare systems and geographical contexts. In addition, mixed-method approaches combining quantitative and qualitative data may provide a deeper understanding of the factors that influence healthcare professionals' responses to IPV. Exploring the effectiveness of specific training interventions may also contribute to improving professional preparedness and strengthening healthcare responses to intimate partner violence.

## 5 Conclusion

The findings of this study indicate that nursing professionals perceive heterogeneous levels of competence in the management of intimate partner violence (IPV). The lowest levels of perceived competence were observed in the dimension related to intervention and referral, suggesting that nurses may feel less prepared to take direct action or initiate referral processes when encountering situations of violence within intimate relationships. In contrast, higher levels of perceived competence were reported in the psychosocial support dimension, reflecting the traditional emphasis of nursing practice on emotional care and patient support.

Professional experience emerged as the only sociodemographic variable significantly associated with perceived competence in certain areas, specifically in the detection and documentation of abuse. Nurses with intermediate levels of professional experience reported higher levels of perceived competence in these dimensions, suggesting that clinical exposure may contribute to the development of these skills over time.

The study also identified the presence of perceptual biases in how healthcare professionals conceptualize intimate partner violence. A large proportion of participants associated IPV primarily with female victims and heterosexual relationships, indicating the persistence of stereotypical representations that may influence professional assessment and potentially limit the identification of other forms of violence within intimate relationships.

Overall, these findings highlight the need for comprehensive training strategies aimed at improving healthcare professionals' preparedness in the detection, documentation, and management of IPV. Addressing both technical competencies and implicit biases may contribute to more effective and inclusive responses to violence within intimate relationships in healthcare settings.

In addition to continuing professional development, integrating training on intimate partner violence into undergraduate nursing education may be essential to ensure early acquisition of competencies in this area.

## 6 Limitations

Several limitations should be considered when interpreting the results of this study. First, the sample was limited to nursing professionals working in the Community of Madrid, which may restrict the generalizability of the findings to other geographical regions or healthcare systems. Second, the study relied on self-reported perceptions of competence, which may not necessarily reflect actual professional behavior in real clinical situations. Third, the cross-sectional design prevents the establishment of causal relationships between professional characteristics and perceived competence.

The use of self-reported measures and online recruitment may introduce self-selection bias. Additionally, the extended data collection period may have introduced temporal variability.

Despite these limitations, the study provides valuable insights into healthcare professionals' perceptions of IPV management and highlights important areas for improvement in professional training.

## 7 Practical implications

The results of this study have important implications for both clinical practice and professional education. Healthcare institutions should consider incorporating structured training programs on intimate partner violence into continuing education initiatives for nursing professionals. Such programs should address not only clinical skills related to detection and intervention but also communication strategies for conducting sensitive interviews with patients.

Additionally, training initiatives should include awareness of potential cognitive biases and stereotypes that may influence the professional interpretation of IPV situations. Promoting inclusive perspectives and improving documentation practices may contribute to better identification of violence cases and more effective coordination with legal and social support services.

## Data Availability

The original contributions presented in the study are included in the article/Supplementary material, further inquiries can be directed to the corresponding author.

## References

[1] Coll-Vinent B Echeverría T Rodríguez D Santiñá M. Violencia intrafamiliar y de género vista por los profesionales de la salud. Medicina Clínica. (2007) 128:317. doi: 10.1016/S0025-7753(07)72572-1 17338868

[2] Plazaola-Castaño J Ruiz Pérez I. Violencia contra la mujer en la pareja y consecuencias en la salud física y psíquica. Medicina Clínica. (2004) 122:461–7. doi: 10.1016/S0025-7753(04)74273-6 15104959

[3] Bugarín-González R Bugarín-Diz C. Aspectos éticos en la atención sanitaria de la violencia de género. SEMERGEN - *Medicina de Familia*. (2014) 40:280–5. doi: 10.1016/j.semerg.2014.03.011 24815861

[4] Fisher CA Rudkin N Withiel TD May A Barson E Allen B . Assisting patients experiencing family violence: a survey of training levels, perceived knowledge, and confidence of clinical staff in a large metropolitan hospital. Women's Health. (2020) 16:1745506520926051. doi: 10.1177/1745506520926051 32716732 PMC7385847

[5] Rojas Loría K Gutiérrez Rosado T Alvarado R Fernández Sánchez A. Actitud hacia la violencia de género de los profesionales de Atención Primaria: estudio comparativo entre Cataluña y Costa Rica. Atención Primaria. (2015) 47:490–7. doi: 10.1016/j.aprim.2014.10.008 25559565 PMC6983685

[6] Fernández Alonso MdC Polo Usaola C Casas Rodríguez P. Impacto de la atención a las víctimas de violencia de género en los y las profesionales de la salud. Atención Primaria. (2024) 56:102856. doi: 10.1016/j.aprim.2023.102856 38310072 PMC11583870

[7] Cantera L Gamero V. La violencia en la pareja a la luz de los estereotipos de género. Psico. (2010) 19:235–46. doi: 10.5093/in2010v19n2a3

[8] Bair-Merritt MH Giardino AP Clements D Webb L. Why do women use intimate partner violence? A systematic review. Trauma, Violence, Abuse. (2010) 11:202–16. doi: 10.1177/1524838010379003 20823071 PMC2994556

[9] Luna MEM Espinosa LMC Ribas JMB Beiras A. Stereotypes of intimate partner violence: do sex and sexual orientation matter? Psicol Teoria E Pesquisa. (2016) 32. doi: 10.1590/0102-3772e323210

[10] Swan SC Gambone LJ Caldwell JE Sullivan TP Snow DL. A review of research on women's use of violence with male intimate partners. Violence Vict. (2008) 23:301–14. doi: 10.1891/0886-6708.23.3.301 18624096 PMC2968709

[11] Aguilera Jiménez A. Violencia de la mujer hacia el hombre, mito o realidad? ReiDoCrea: Revista electrónica de investigación Docencia Creativa. (2015). Available online at: http://hdl.handle.net/10481/34597

[12] Rojas-Andrade R Galleguillos G Miranda P Valencia J. Los hombres también sufren. Estudio cualitativo de la violencia de la mujer hacia el hombre en el contexto de pareja. Rev Vanguardia Psicol Clí*n Teor Práct*. (2013) 3:150–9.

[13] Gasch-Gallén Rodríguez-Arenas M Tomás-Aznar C Latasa P Gil-Borrelli CC Velasco-Muñoz C . Inclusión de la orientación afectivo-sexual y de las identidades de género como determinantes sociales de la salud. Gaceta Sanitaria. (2018) 32:400. doi: 10.1016/j.gaceta.2017.12.008 29628122

[14] Obón-Azuara B Gasch-Gallén Gutiérrez-Cía I Tomás-Aznar C. Políticas sanitarias, perspectiva de género y diversidad afectivo sexual: una asignatura pendiente? Atención Primaria. (2020) 52:123–5. doi: 10.1016/j.aprim.2019.06.004 31387764 PMC7025967

[15] Garcia-Esteve L Torres A Navarro P Ascaso C Imaz ML Herreras Z . Validación y comparación de cuatro instrumentos para la detección de la violencia de pareja en el ámbito sanitario. Med Clín. (2011) 137:390–7. doi: 10.1016/j.medcli.2010.11.038 21757210

[16] Asensi Pérez LF Flores Fernández E Nevado Duarte K. Evaluación pericial psicológico-forense del trastorno por estrés postraumático complejo en víctimas de violencia de género. Rev Española Med Legal. (2024) 50:76–81. doi: 10.1016/j.reml.2023.09.001

[17] Ross R Roller CG Rusk T Martsolf DS Draucker CB. The SATELLITE sexual violence assessment and care guide. Women's Health Care. (2009) 8:25–31. 22506255 PMC3324818

[18] Ross R Draucker CB Martsolf DS Adamle K Chiang-Hanisko L Lewandowski W. The bridge: providing nursing care for survivors of sexual violence. J Am Acad Nurse Pract. (2010) 22:376–82. doi: 10.1111/j.1745-7599.2010.00519.x 20590958

[19] Borrell-Carrio F Epstein RM Pardell Alentá H. Profesionalidad y professionalism: fundamentos, contenidos, praxis y docencia. Medicina Clínica. (2006) 127:337–42. doi: 10.1157/13092322 16987454

[20] Ross R Stidham AW Saenyakul P Creswell JW. Intimate partner violence, emotional support, and health outcomes among Thai women: a mixed methods study. J Royal Thai Army Nurses. (2015) 16:14–24.

[21] Noriega N Juarros-Basterretxea J Herrero J. Implicación de los profesionales de la salud en los casos de violencia en la pareja contra la mujer: La influencia de las actitudes sexistas hacia la mujer. Rev Iberoamericana Psicol Salud. (2020) 11:31–41. doi: 10.23923/j.rips.2020.01.033

[22] Ross R Sheppard F Almotairy M Hirst J Jenkins M. Pilot study of the SATELLITE on nurses' knowledge and confidence toward assessing and caring for female victims of sexual violence. Nurs Rep. (2024) 14:1287–96. doi: 10.3390/nursrep14020097 38804430 PMC11130817

[23] Aguilera Jiménez A Barba Priego M Fuentes Gutiérrez M López Molina E Villacreces Flores NM García Ramírez JM. Violencia de la mujer hacia el hombre, mito o realidad? Violence of women against men, myth or reality? (2015). doi: 10.30827/Digibug.34597

