# Supplementary material for: Perceived competence and cognitive bias in nurses' assessment of intimate partner violence: a cross-sectional study
Source: Front Public Health. 2026 May 7;14:1835799. doi: 10.3389/fpubh.2026.1835799 (PMC13190558; doi:10.3389/fpubh.2026.1835799)
Supplement: Supplementary file 1 [file Data_Sheet_1.zip › Supplementary Files/Nursing Activities Assessment Scale.pdf]

Nursing Activities Assessment Scale (NIC: 6403) for Intimate Partner Violence

First of all, thank you for completing this scale. It is part of my PhD thesis in Nursing on intimate partner violence at Universidad CEU San Pablo. The scale is anonymous and takes less than 5 minutes to complete.

This scale aims to identify high-risk domestic dependency relationships and actions to prevent further physical, sexual, emotional harm, or exploitation of one partner by the other. It is designed to assess the ability of nursing staff to detect intimate partner violence in both men and women.

Principal Investigator

david.caserobenavente@ceu.es

INSTRUCTIONS

Please indicate in each item your level of personal ability/difficulty in performing the following activities in daily clinical practice.

INFORMED CONSENT

I, being of legal age and fully capable, expressly consent to participate in this anonymous online health-related scale.

I have been informed that the purpose of this scale is to assess nursing practice in the context of intimate partner violence, and that the results will be used for non-profit research purposes related to the completion and publication of a doctoral thesis.

I understand that the scale is anonymous, and therefore my personal data will not be recorded or used at any time. Participation is voluntary, and I may withdraw my consent at any time without any consequences for my health or rights. I have also been informed that participation in this scale entails no risk to my health.

Accordingly, I freely and explicitly give my informed consent to participate in this anonymous online health-related scale.

This informed consent complies with the requirements established by Organic Law 3/2018 of 5 December on the Protection of Personal Data and Guarantee of Digital Rights (LOPDGDD).

When you submit this form, your personal details (such as name or email address) will not be collected unless you provide them voluntarily.

Sociodemographic Variables

1. Sex

* Male
* Female
* Prefer not to say

2. Age

* 20–30 years
* 31–40 years
* 41–50 years
* 50 years

3. Education level

* Diploma
* Bachelor’s degree
* Postgraduate
* Doctorate

4. Years of professional experience

* <5 years
* 6–10 years
* 11–15 years
* 16 years

5. Main professional role as a nurse

* Primary care
* Hospital care
* Out-of-hospital care
* Teaching
* Social care (nursing homes)
* School nursing

Scale Items (Likert 1–5)

(1 = Not competent at all; 5 = Highly competent)

6. Observe signs and symptoms of physical abuse

(e.g., multiple injuries at different stages of healing, lacerations, bruises without explanation, alopecia areas, ligature marks, defensive wounds, human bite marks).

7. Observe signs and symptoms of sexual abuse

(e.g., presence of semen or dried blood, genital injuries, sexually transmitted infections, behavioral changes of unclear origin).

8. Observe signs and symptoms of exploitation

(e.g., inadequate provision of basic needs despite sufficient resources, deprivation of personal belongings).

9. Observe signs and symptoms of emotional abuse

(e.g., low self-esteem, depression, humiliation, excessive caution toward partner, self-harm, suicidal ideation).

10. Observe excessive submissiveness (e.g., passive compliance with hospital procedures).

11. Observe and document partner interactions

(e.g., frequency/duration of visits, exaggerated or insufficient reactions).

12. Observe progressive deterioration in physical condition.

13. Observe progressive deterioration in emotional condition.

14. Observe repeated visits to healthcare services for minor complaints.

15. Observe use of community resources.

16. Document evidence of abuse using standardized tools and photographs.

17. Listen attentively when the person begins to talk about their problems.

18. Assess risk factors associated with domestic abuse

(e.g., history of violence, substance abuse, depression, social isolation, pregnancy, poverty, unemployment).

19. Assess history of abuse-related symptoms

(e.g., chronic pain, PTSD, anxiety, depression).

20. Identify inconsistencies in explanations of injuries.

21. Determine correlation between injury type and reported cause.

22. Interview the patient and/or others privately (without partner present).

23. Encourage hospital admission when appropriate.

24. Establish a system to flag suspected abuse cases in medical records.

25. Encourage expression of feelings (fear, guilt, shame).

26. Provide positive reinforcement regarding self-worth.

27. Support victims in taking protective actions.

28. Assist individuals/families in coping strategies.

29. Help evaluate strengths and weaknesses in relationships.

30. Refer at-risk individuals to appropriate services
(e.g., public health, social services, legal support).

31. Refer abusive partners to appropriate services.

32. Provide confidential information about shelters.

33. Initiate safety planning.

34. Promote community education programs.

35. Report suspected abuse according to legal requirements.

Final Questions

36. What is the main barrier (in your experience) to performing these nursing activities?

* Consultation time
* Training
* Personal resistance to the topic
* Patient-related factors and interviewing skills

37. While completing the scale, I mainly thought about the victim as:

* Woman

* Man

* Both

38. While completing the scale, I thought about the relationship as:

* Heterosexual

* Homosexual

* Both
