# Supplementary material for: Perceived competence and cognitive bias in nurses' assessment of intimate partner violence: a cross-sectional study
Source: Front Public Health. 2026 May 7;14:1835799. doi: 10.3389/fpubh.2026.1835799 (PMC13190558; doi:10.3389/fpubh.2026.1835799)
Supplement: Supplementary file 1 [file Data_Sheet_1.zip › Supplementary Files/Escala de valoracio╠ün de actividades enfermeras (NIC: 6403), frente a la violencia de pareja.pdf]

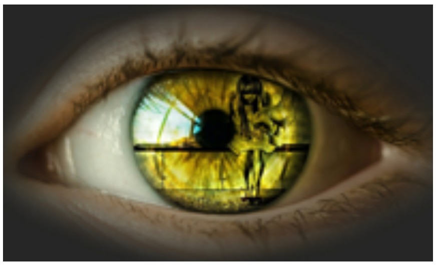

Escala de valoración de actividades enfermeras (NIC: 6403), frente a la violencia de pareja

LO PRIMERO DAROS LAS GRACIAS POR RELLENAR LA ESCALA, FORMA PARTE DE MI TESIS DE DOCTORADO EN ENFERMERIA EN VIOLENCIA PAREJA, UNIVERSIDAD CEU SAN PABLO. LA ESCALA ES ANÓNIMA Y SE REALIZA EN MENOS DE 5 MINUTOS.
Identificación de las relaciones de dependencia domésticas de alto riesgo y de las acciones para prevenir que se inflija un mayor daño físico, sexual, emocional o la explotación de uno de los miembros de la pareja. Esta escala pretende valorar la detección en ambos sexos la violencia de pareja por parte del personal de enfermería.

Investigador principal
david.caserobenavente@ceu.es

INSTRUCCIONES
Por favor indicar en cada Ítem el grado capacidad/ dificultad personal para realizar las siguientes actividades en la consulta diaria.

CONSENTIMIENTO INFORMADO
Yo, mayor de edad y con plena capacidad para obrar, consiento expresamente en participar en la escala anónima online de carácter sanitario que se me ofrece dando mi consentimiento al realizarla.
He sido informado de que la escala tiene como objetivo valorar la praxis enfermera en violencia intrafamiliar, y que los resultados se utilizarán con fines de investigación sin ánimo de lucro para la realización y publicación de la tesis doctoral. He sido informado de que la escala es anónima, por lo que mis datos personales no serán registrados, ni utilizados en ningún momento. He sido informado de que la participación en la escala es voluntaria y que puedo retirar mi consentimiento en cualquier momento, sin que ello tenga repercusiones para mi salud ni para mis derechos. He sido informado de que la escala no tiene ningún riesgo para mi salud.
En consecuencia, otorgo mi consentimiento libre, informado y expreso para participar en la escala anónima online de carácter sanitario que se me ofrece. Este consentimiento informado cumple con los requisitos establecidos en la Ley Orgánica 3/2018, de 5 de diciembre, de Protección de Datos Personales y garantía de los derechos digitales (LOPDGDD).

Cuando envíe este formulario, no recopilará automáticamente sus detalles, como el nombre y la dirección de correo electrónico, a menos que lo proporcione usted mismo.

* Obligatorio

1. SEXO *

- HOMBRE
- MUJER
- PREFIERO NO DECIRLO

2. EDAD *

- 20-30 AÑOS
- 31- 40 AÑOS
- 40-50 AÑOS
- +50 AÑOS

3. FORMACION *

- DIPLOMADO
- GRADO
- POSTGRADO
- DOCTORADO

4. AÑOS TRABAJADOS *

- MENOS DE 5 AÑOS
- ENTRE 6 -10 AÑOS
- ENTRE 11-15 AÑOS
- + DE 16 AÑOS

5. OCUPACIÓN LABORAL PRINCIPAL COMO ENFERMERA *

- ATENCIÓN PRIMARIA
- ATENCIÓN HOSPITALARIA
- ATENCIÓN EXTRAHOSPITALARIA
- DOCENCIA
- ASISTENCIA SOCIAL (RESIDENCIAS)
- ENFERMERA ESCOLAR

6. **Observar si hay signos y síntomas de abuso físico**
(p. ej., numerosas lesiones en distintas etapas de curación, laceraciones, hematomas o contusiones sin explicación, zonas sin cabellos en la cabeza, marcas de ataduras en las muñecas o tobillos; contusiones «defensivas» en los antebrazos y marcas de mordeduras humanas). *

Nada capacitado.

Muy capacitado

1	2	3	4	5

7. **Observar si hay signos y síntomas de abuso sexual** (p. ej., presencia de semen o de sangre seca, lesiones en los genitales externos, enfermedades de transmisión sexual, o comportamiento dramático o cambios de salud de etiología indeterminada). *

Nada capacitado.
Muy capacitado

1 2 3 4 5

8. **Observar si hay signos y síntomas de explotación** (p. ej., provisión inadecuada de las necesidades básicas cuando se dispone de recursos adecuados, privación de posesiones personales, pérdida inexplicada de la ayuda *

Nada capacitado
Muy capacitado

1 2 3 4 5

9. **Observar si hay signos y síntomas de abuso emocional** (p. ej., baja autoestima, depresión, humillación y sentimientos de derrota; comportamiento demasiado cauteloso respecto a la pareja; autoagresión o actitudes suicidas). *

Nada capacitado
Muy capacitado

1 2 3 4 5

10. **Observar si el individuo muestra una excesiva sumisión, tal como someterse de forma pasiva a los procedimientos del hospital.** *

Nada capacitado
Muy capacitado

1 2 3 4 5

11. **Observar las interacciones de la pareja y registrarlas, según corresponda**
(p.ej., registrar las horas y la duración de las visitas de la pareja durante la hospitalización, reacciones insuficientes o exageradas por parte de la pareja). *

Nada capacitado
Muy capacitado

1	2	3	4	5

12. **Observar si hay un deterioro progresivo del estado físico.** *

Nada capacitado
Muy capacitado

1	2	3	4	5

13. **Observar si hay un deterioro progresivo del estado emocional de losindividuos.** *

Nada capacitado
Muy capacitado

1	2	3	4	5

14. **Observar si se repiten las visitas a clínicas, urgencias o consultas al médico por problemas leves.** *

Nada capacitado
Muy capacitado

1	2	3	4	5

15. **Observar el uso de recursos de la comunidad.** *

Nada capacitado
Muy capacitado

1 2 3 4 5

16. **Documentar la evidencia de abusos físicos o sexuales utilizando herramientas estandarizadas de valoración y fotografías.** *

Nada capacitado
Muy capacitado

1 2 3 4 5

17. **Escuchar atentamente a la persona que empieza a hablar acerca de sus propios problemas.** *

Nada capacitado
Muy capacitado

1 2 3 4 5

18. **Investigar si existen factores de riesgo asociados con el abuso doméstico**
(p. ej., historial de violencia doméstica, abusos, rechazo, exceso de crítica o sentimientos de inutilidad y falta de amor; necesidad elevada de cuidado físico; muchas responsabilidades de cuidado familiar; consumo de sustancias; depresión; enfermedades psiquiátricas graves; aislamiento social; embarazo; pobreza; desempleo; dependencia económica; falta de hogar; infidelidad; divorcio, o muerte de un ser querido). *

Nada capacitado
Muy capacitado

1 2 3 4 5

19. **Investigar si existe historial de síntomas de abuso doméstico** (p. ej.,numerosas lesiones accidentales, múltiples síntomas somáticos, dolor abdominal crónico, cefaleas crónicas, dolor pélvico, ansiedad, depresión, síndrome de estrés postraumático y otras alteraciones psiquiátricas). *

Nada capacitado
Muy capacitado

1 2 3 4 5

20. **Identificar incongruencias en la explicación de la causa de las lesiones.** *

Nada capacitado
Muy capacitado

1 2 3 4 5

21. **Determinar la correlación entre el tipo de lesión y la descripción de la causa.** *

Nada capacitado
Muy capacitado

1 2 3 4 5

22. **Entrevistar al paciente y/o a alguna otra persona que conozca la situación acerca del presunto abuso en ausencia de la pareja.** *

Nada capacitado
Muy capacitado

1 2 3 4 5

23. **Alentar el ingreso del paciente para una mejor observación y estudio, cuando corresponda.** *

Nada capacitado
Muy capacitado

1	2	3	4	5

24. **Establecer un sistema para marcar las historias clínicas individuales en las que existan sospechas de abuso.** *

Nada capacitado
Muy capacitado

1	2	3	4	5

25. **Animar la expresión de preocupaciones y sentimientos incluidos: el miedo, culpabilidad, vergüenza y auto culpabilidad.** *

Nada capacitado
Muy capacitado

1	2	3	4	5

26. **Proporcionar afirmaciones positivas sobre la valía.** *

Nada capacitado
Muy capacitado

1	2	3	4	5

27. **Proporcionar apoyo para que las víctimas tomen medidas y realicen cambios para evitar represalias posteriores.** *

Nada capacitado
Muy capacitado

1 2 3 4 5

28. **Ayudar a los individuos y a sus familias a desarrollar estrategias de afrontamiento frente a situaciones estresantes.** *

Nada capacitado
Muy capacitado

1 2 3 4 5

29. **Ayudar a los individuos y a sus familias a evaluar objetivamente los puntos fuertes y débiles de las relaciones.** *

Nada capacitado
Muy capacitado

1 2 3 4 5

30. **Remitir a los individuos con riesgo de abuso o a aquellos que los hayan sufrido a especialistas y servicios apropiados** (p. ej., servicios de salud pública, servicios sociales, asesoramiento, asistencia legal) *

Nada capacitado
Muy capacitado

1 2 3 4 5

31. **Remitir al cónyuge que abusa a especialistas y servicios apropiados.** *

Nada capacitado
Muy capacitado

1 2 3 4 5

32. **Proporcionar información confidencial respecto a los albergues para personas que sufren violencia doméstica, según corresponda.** *

Nada capacitado
Muy capacitado

1 2 3 4 5

33. **Iniciar el desarrollo de un plan de seguridad para utilizar si la violencia se intensifica.** *

Nada capacitado
Muy capacitado

1 2 3 4 5

34. **Iniciar programas de educación de la comunidad diseñados para disminuir la violencia.** *

Nada capacitado
Muy capacitado

1 2 3 4 5
