# Supplementary figures and images for: Perceived competence and cognitive bias in nurses' assessment of intimate partner violence: a cross-sectional study
Source: Front Public Health. 2026 May 7;14:1835799. doi: 10.3389/fpubh.2026.1835799 (PMC13190558; doi:10.3389/fpubh.2026.1835799)

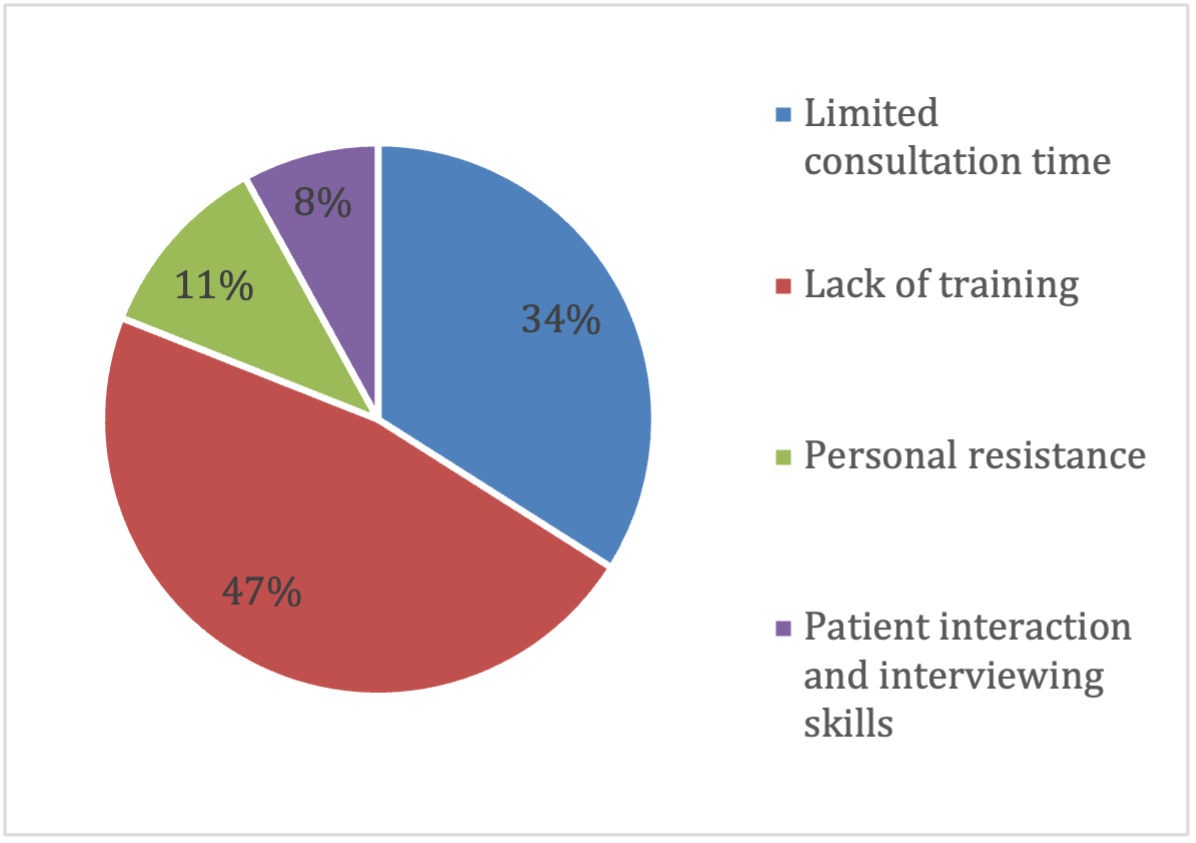

Supplement: Supplementary file 1 [file Data_Sheet_1.zip › Supplementary Files/Supplementary Figure 1.png]
